# Hedging climate change risks in Southern Africa’s agricultural industry using catastrophe bonds

**DOI:** 10.4102/jamba.v16i1.1641

**Published:** 2024-08-12

**Authors:** Thomas Mutsvene, Heinz E. Klingelhöfer

**Affiliations:** 1 Department of Finance and Investment, Faculty of Economics and Finance, Tshwane University of Technology, Pretoria, South Africa

**Keywords:** agriculture, catastrophe bonds, climate-change risk, disaster-hedging, risk, underwriting capacity, CAT bond market

## Abstract

The agricultural sector is one of the key economic contributors in Southern Africa. However, agricultural production has been highly affected by climate change risks such as tropical cyclones, floods, droughts, heatwaves, hail, etc., which threaten food and nutrition security, livelihoods and business sustainability. Because of underwriting capacity problems, insurers and reinsurers have failed to provide cover for climate change risks. Also, derivatives have failed to provide a reliable option for hedging such risks. This paper explores the concept of catastrophe bonds (CAT bonds) in providing climate change risk finance. Employing the content analysis, the research shows how CAT bonds can help traditional (re)insurance in providing sufficient hedge against climate change risks and in improving disaster-preparedness, disaster risk-reduction, post-recovery initiatives and sustainable socio-economic agricultural development. The findings show that the adoption of CAT bonds can improve (re)insurers´ underwriting capacity and may enhance agricultural land policies, development, food and nutrition security and employment.

**Contribution:**

The paper shows how CAT bonds can be employed to hedge against climate change risks in agricultural production and to increase (re)insurers´ underwriting capacity. It further discusses the attractiveness of CAT bonds as another investment option for agricultural investors and how to develop and institutionalise a CAT bond market.

## Introduction

Southern Africa has been known for years as the breadbasket of the African continent. The total agricultural production of South Africa, Zimbabwe, Mozambique, Botswana and Zambia had been highly significant to the total continental production until climate change-induced disasters became a common experience in the new millennium (Westermann 2019). Since 2000, there has been a spat of climate shocks in the form of tropical cyclones, droughts and floods, which caused huge agricultural losses beyond the sums insured and insurers’ underwriting capacity. Catastrophic (CAT) events have posed significant covariate risks to small and least developed countries (Zimmerman & Carter 2003:1). The Insurance Information Institute (III) 2019 Report highlighted that the overall losses from worldwide natural catastrophes in 2019 totalled US$150 billion of which only US$52bn losses were insured (III 2019). The Allianz Risk Barometer 2022 First Quarter (Q1) Report further indicated that according to Swiss Re, the annual insured natural catastrophe losses reached US$105bn out of US$252.1bn economic losses in 2021, the fourth highest record since 1970 (Allianz 2022). In 2023, global economic losses from climate change disasters reached US$380bn, only US $118bn of which were insured while US$262bn remained uninsured because of underwriting capacity problems (AoN 2024).

In the same way, Southern Africa experienced several catastrophes in recent years, leaving devastating effects across the region: The rate of increase in annual natural climate change catastrophe losses is significantly rising and worrisome. Zimbabwe, Mozambique and Malawi were hit by cyclone Idai in 2019. It claimed at least 1303 lives, affected more than 3 million others in Zimbabwe and destroyed agricultural production of more than 500 000 ha of crops in Mozambique alone, estimated to be a US $513m loss (FAO 2019; IPS 2019; Westermann 2019). In April and May 2022, South Africa’s KwaZulu Natal and Eastern Cape provinces were ravaged by floods, claiming 443 lives, leaving approximately 48 unaccounted for and affecting an estimated 1 386 941 hectares of agricultural cropland (Government of South Africa [GoSA] 2022). Besides this, 4000 houses were destroyed, 8300 others partially damaged and over 40 000 people displaced by the floods in April alone (GoSA ECHO Report 2022).

Regular geospatial information for vulnerability and impact assessment in support of climate risk preparedness and response programmes takes time to be updated (GoSA ECHO Report 2022; Weber et al. 2015). Therefore, all this necessitates the need to find a solution at least to the financial consequences of the climate change risks that are seemingly becoming ubiquitous in Southern Africa. The traditional insurance and risk finance mechanisms are falling short of covering these risks with most insurers undercapitalised to underwrite climate risks, while regional reinsurers have never teamed up to provide cover either (Götze & Gürtler 2022). Considering environmental factors such as climate change mitigation and adaptation, preservation of biodiversity and prevention of pollution, as well as the circular economy, guaranteed future returns are important for many investment decisions, particularly in agriculture (FAO 2019). Hedging against agricultural risks and hard times, regardless of the magnitude and severity, in Southern Africa has commonly relied on a mix of instruments that may include insurance, derivatives, investments into mitigation and government bailouts (Steve 2019).

Financial instruments like derivative contracts in the form of futures and forward contracts, swaps as well as option financing have been in use for a long time (McGuigan et al. 2012). However, employing such derivatives to hedge climate change risks is highly risky because of the hazards of trading these financial instruments over the counter with individual financial institutions and the lack of standard regulation in trading and clearing contracts, which makes it difficult to get the contracts fulfilled when a contract party fails (McGuigan et al. 2012). Therefore, ‘normal’ derivatives are far from being ideal when it comes to insuring agriculture against catastrophe risks triggered by climate change. In addition, the uncertainty of the time a climate change-triggered risk may occur, coupled with its frequency, makes put options, swaps or futures contracts difficult to use. If one party to the contract fails during the period of catastrophe risks, it becomes difficult to finance the risk. This leaves a lot of catastrophe risks difficult to be hedged using derivatives, leaving the government exposed to providing support during and post-disaster periods.

As derivatives are not commonly used in Southern Africa, insurance remains as the most popular risk financing mechanism in the agricultural sector (Steve 2019). However, the challenge of insurance is that it covers general risks, but most policies exclude climate change risks such as tropical cyclones, floods, strong winds, hurricanes, tsunamis, tornados and so on (Weber et al. 2015). This is because most of the insurers lack underwriting capacity for high-severity risks such as climate change-triggered agricultural risks. Furthermore, risk transfer mechanisms to insurance companies in expectation of indemnity are falling short as climate change is triggering high-severity risks such as tropical cyclones, floods and strong winds that are leaving trails of destruction beyond the underwriting capacity of insurers, co-insurers and reinsurers. The resulting agricultural risks threaten crops, livestock, property and equipment as well as human life. Should a climate change catastrophe risk strike, insurers’ premium pools will be eroded, leaving the insurers with no other option than to close their doors. Hence, while, on the one hand, there are some overambitious insurers and reinsurers who try to develop insurance cover for these risks, on the other hand, their balance sheets scare them from accepting catastrophe risk business, especially in the agricultural sector. Therefore, many of these financial players end up rejecting to insure climatic change-triggered risks.

Because of this increased exposure, the interdependence between the Southern African Development Community and good climate in boosting agricultural reserves is under severe threat from climate change risks. While the current method(s) used to manage climate risks in the agricultural sector such as insurance and property or commodity hedging may have proved to work in the past, because of low underwriting capacities and the described hazards of employing derivatives to hedge climate change risks, they are falling short to match the magnitude of loss or damage caused by catastrophe risks. Therefore, urgent adaptation to climate change and the risks it triggers has become a necessity rather than a want. For this purpose, this paper intends to propose the concept of using catastrophe bonds to hedge against climate change risks in Southern Africa’s agricultural sectors.

The paper, therefore, seeks to achieve the following objectives:

- propose the adoption of catastrophe bonds (CAT bonds) as a new way of financing climate change risks in Southern Africa’s agricultural sector
- demonstrate the establishment and institutionalisation of a CAT bond market for agricultural risks in Southern Africa
- show the managerial implications of using CAT bonds over other agricultural risk hedging techniques in Southern Africa.

## Research methodology

The research is a desktop study following content analysis of journals, articles and annual reports of (re)insurers for the period 2012–2023. This period was selected to use more current information on CAT bonds and climate change risk management. By reviewing the literature on catastrophe bonds using keywords such as ‘catastrophe bonds’, ‘disaster hedging’, ‘climate change risk’ and ‘underwriting capacity’, the study examines whether adopting CAT bonds may be a way to finance and hedge climate change risks for the agricultural sectors in Southern Africa. An index formula is developed to structure CAT bonds operations using predefined trigger thresholds and bond underwriting exhaustion points that make the financial instrument attractive enough to speculative investors. Focusing on the Southern African region, some examples are taken from six randomly selected countries, namely South Africa, Zimbabwe, Mozambique, Zambia, Botswana and Namibia, to conceptually model trigger levels structuring for climate change risks. As all the data and information used in this research are publicly available, hence, there is no need for ethical clearance as no ethical risks are foreseen.

## Literature review

Literature on the use of catastrophe bonds (CAT bonds) in various Southern African economic sectors including agriculture, is still in its infancy. The recently increased climate change risks affecting the agricultural sector require new risk hedging strategies as traditional insurance is failing to cope with the huge financial risks resulting from climate change. Hence, by reviewing the literature on catastrophe bonds, it shall be examined whether adopting CAT bonds may assist in financing these risks for the agricultural sector in Southern Africa.

### Accepting catastrophe bonds (CAT bonds) in financing climate change risks for the agricultural sector in Southern Africa

The paper investigates the concept of hedging climate change risks using catastrophe bonds (CAT bonds) in Southern Africa’s agricultural sector. Well-structured CAT bonds can mitigate climate change-induced agricultural risks that insurance and hedge instruments like derivatives cannot finance (Miranda & Farrin 2012; Weber et al. 2015). Catastrophic bonds are high-yield debt instruments designed to raise funds for governments or companies in the insurance industry in times of natural disasters by allowing issuers to receive funding from the bond only if specific conditions such as floods, cyclones, tornadoes or earthquakes occur (Cummins & Weiss 2009). In a similar sense, already Kirk and Pieterse (2019), in their closely related exploratory study on the viability of pandemic CAT bonds in South Africa, recommended the use of CAT bonds to reduce insurers’ and governments’ exposure during CAT events like the pandemics. Drifting from the use of insurance and derivatives in hedging CAT events like climate change risks in the agricultural sector to the adoption of catastrophe bonds can be done using the disaster risk management (DRM) process.

**FIGURE 1:**
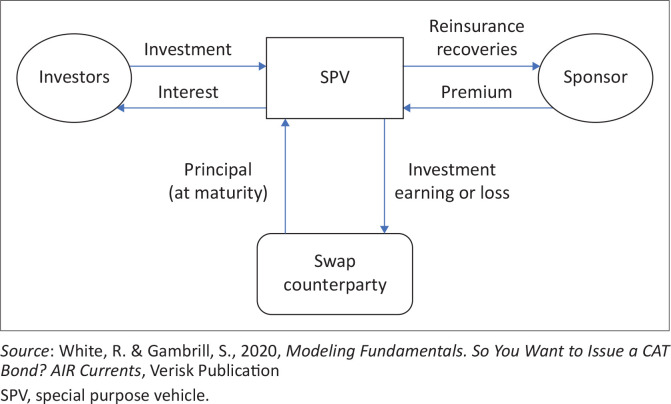
Flow of catastrophic bond finances/structure of a catastrophic bond transaction.

Different from normal bonds and derivatives, CAT bonds are insurance-linked securities (ILS) (Ando et al. 2022). They are used as a method of transferring insurance risk to capital markets where their proceeds are invested in nearly risk-free to risk-free assets. In this way, they often generate money market returns and complement the insurer’s premium, thereby solving underwriting capacity issues. This allows the CAT bond to pay a quarterly coupon to the investor with usually a substantial spread above market returns for bonds with equivalent ratings as investors ‘appear to earn both a liquidity and a “novelty” premium for taking insurance risk’ (Risk Management Solutions 2012:6–7). To do so, this requires a sponsoring or ceding insurer to establish a special purpose vehicle (SPV) as explained by employing Figure 1.

This SPV would create a reinsurance agreement with the sponsoring/ceding insurer in a tax efficient jurisdiction. It will also issue a note to investors with default provisions that mirror the terms of the reinsurance agreement, containing trigger events (White & Gambrill 2020). The funds generated from the sale of the note are managed in a separate collateral account where they generate money market returns, which consolidate the insurer’s premiums to cover the agricultural risk/triggered event. Part of the investment funds kept in the collateral account by the SPV can be invested in short-term financial instruments like Treasury Bills (TBs) and Bankers Acceptances (BAs) held by a SWAP counterparty, which generates quicker money market returns. These money market returns can help the SPV in paying the agriculture CAT bond speculative investors’ coupon as well as contributing to reinsurance recoveries requested by the sponsor. The trigger mechanisms need to be balanced to accommodate preferences of the sponsor and the investor. The use of CAT bonds in hedging against climate change risks in the agriculture sector can be done following the DRM process to determine the stage requiring financing and trigger points (Götze & Gürtler 2022; Westermann 2019).

**FIGURE 2:**
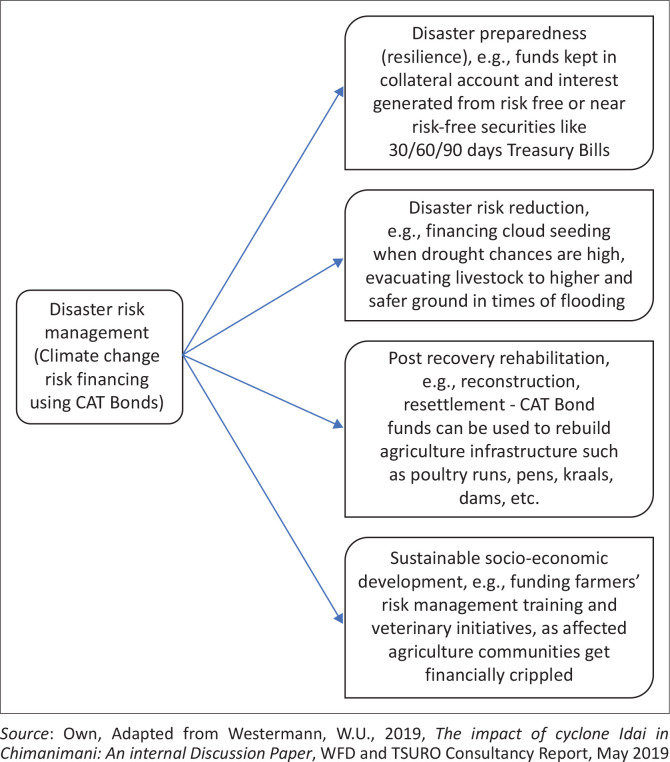
Disaster risk management concept/process.

Reinforcing the DRM process, which shows possible responses to agricultural risks that can be implemented at any stage during post-climate disaster recovery and development, can be more effective using CAT bonds (Mutsvene & Klingelhöfer 2024). CAT bonds provide a proactive risk preparedness, response and post-recovery concept necessary for effective agricultural production projects and programs as shown in Figure 2 (AoN 2024).

Given the above concept of DRM, the adoption of catastrophe bonds (CAT bonds) can stimulate every stage of the DRM concept (namely disaster preparedness (resilience), disaster risk reduction, post recovery rehabilitation, and sustainable socio-economic development), so that climate change risks in Southern Africa’s agricultural sectors can be proactively managed: Funds generated through CAT bond subscriptions allow for reinforced disaster preparedness as well as the implementation of risk reduction techniques – and by that incentivise production. The post-disaster recovery, rehabilitation and reconstruction phases will become much easier as funds generated from investors’ principal amounts may be channelled to the affected farmers and communities, putting them into the same pecuniary positions as before the agricultural loss. Therefore, instead of relying on their own underwriting capacity, the insurers/reinsurers will collect these funds from the SPV’s collateral account as per the underwritten terms of the agriculture CAT bond and set trigger levels (Van Wyk 2021).

To make the CAT bond attractive enough to become a tool to cover agricultural risks, the trigger mechanisms for the payout of the bond need to be balanced to accommodate the preferences of the sponsor and the investor. Continuing the thought mentioned in the previous paragraph on CAT bond underwriting terms and trigger levels, a trigger may specify, for example, that the payout from an agricultural flood CAT bond is based on actual losses to the sponsor (who will then use the money to pay e.g. the farmers). This will make the agricultural CAT bond attractive to the insurer, who does not need to have the underwriting capacity, as well as to the farmers, who are threatened by existential risks from climate change and who will value the elimination of basis risk, allowing them to continue with their beneficial agricultural activities without worrying too much about circumstances they cannot influence. ‘Basis risk’ in this context means the difference between the insurers’ (as the sponsor) and farmers’ losses on the one side, and the CAT bond’s payout on the other side when an insured agricultural risk is triggered (White & Gambrill 2020).

In determining the trigger types and protection levels through an iterative process with the sponsors, in order to cover the farmers, a structuring agent may assist (Ando et al. 2022). Employing an iterative process makes this concept adaptable to requirement changes throughout the development of the agricultural CAT bond’s protection level and trigger types. On this basis and depending on the kind of trigger, an Agric CAT bond can then be designed in four different ways, namely:

- Indemnity triggers make agricultural loss recoveries based on the sponsors’/farmers’ actual loss exposure.
- Normal loss triggers involve recreating actual climate change event parameters such as floods, tropical cyclones, hail, drought, etc. into the catastrophe model. These shall help to estimate the financial impact on the portfolio of exposure that was originally used in the estimation of the CAT bond’s risk (regardless of whether the portfolio is similar or not to the sponsor’s actual exposure).
- Industry loss index triggers are based on actual agricultural losses to the entire insurance industry.
- Parametric triggers are based on objective physical characteristics of the agricultural risk.
- Hybrid triggers are a combination of several triggers mentioned before and are used in the issuance of hybrid agricultural CAT bonds (Cummins & Weiss 2009). Hence, they are most suitable for a wide coverage of various climate change risks affecting the agricultural sector – a characteristic that helps in providing cover for most of the risks excluded from insurance policies and other hedging options (Barrieu & Louberge 2009; Van Wyk 2021).^1^

On this basis, an agriculture CAT bond can be customised to have different event parameters such as flood, cyclone, wind, hail and drought being covered. Applying the different triggers introduced above may lead to the following modelling as shown in Table 1.

**TABLE 1:** Authors’ model of agricultural catastrophic bond trigger level structuring for climate change risks.

Type of agricultural CAT bond	Triggers and event parameters	Level (examples)
(1) Flood CAT bond	Indemnity trigger (e.g. CAT event parameter – Flood)	Minimum Destruction of 300 ha of crops or min destruction cost of R50, m whichever happens first and damage in KwaZulu Natal (KZN) capital, South Africa, from 11 April – 01 May 2022. Death or bodily injuries to livestock of at least R15m within 1 week of flooding in KZN. Agric Equipment & Property Damage of at least R20m within a week of flooding in KZN.
(2) Cyclone CAT bond	Parametric trigger (e.g. CAT event parameter – Cyclone)	Minimum of 200mm rainfall, minimum destruction of R15m within radius from 50km radius of Chimanimani, Zimbabwe or Continuous rainfall with strong winds causing economic losses of at least 1bn meticais in Cabo Delgrado province of Mozambique; or Livestock deaths of minimum R6m in farms surrounding Eastern Cape’s Cradock town.
(3) Wind or hurricane CAT bond	Industry loss trigger (e.g. CAT event parameter – Wind/Hurricane)	Minimum windspeed of 90 knots causing an aggregate loss of at least 200 kwachas in Zambia.
(4) Hail CAT bond	Indemnity trigger (e.g. CAT event parameter – Hail)	Leaf crop or fruit/tuber damage of at least R7.5m in Gauteng between 01–15 June 2023.
(5) Drought CAT bond	Normal Loss Trigger (e.g. normal loss parameter – Drought in low rainfall regions/provinces	Annual drought induced agricultural loss in predetermined low rainfall areas of a minimum of 10m Pula in Botswana.
(6) All-risks CAT bond (Hybrid agricultural CAT bond)	Can be a hybrid trigger (a combination of parametric, indemnity, and industry loss triggers depending on stated CAT risks) Any climate risk affecting agriculture like hurricanes, droughts, cyclones, floods, hail, etc.	Minimum destruction on crops, livestock, farm equipment and property of +N$950m in Namibia.

CAT, Catastrophic.

To establish trigger levels for event parameters as mentioned in Table 1, a calculation that combines all parameter readings and recordings is done. This requires that the recordings relate to the CAT event covered by the bond. Then, an index formula may help to solve the confusion arising in determining whether a CAT bond has been triggered by a CAT event or not. Therefore, at the issuance of the agricultural risk CAT bond, the structuring agent will define weights *w*_i_ for each of the CAT event parameter levels *p*_i_:

*w*_i_: Predefined weights in relation to the distribution of the sponsor’s exposure,

*p*_i_: agricultural CAT event parameter (flood, cyclone, hail or wind, etc.) measurement at each of x recording stations.

Using the index formula: *I* = *f* (*w, p*)

The index value *I* calculated can then be compared to the pre-defined trigger level *I*_Thresh_. If the index is above the predefined trigger level *I*_Thresh_ (seen as a threshold), then the agricultural CAT bond is triggered. If it is even beyond the exhaustion threshold *I*_Exh_, the CAT bond is exhausted, and the investors lose the invested principal completely (Mutsvene & Klingelhöfer 2024; Polacek 2018).

### Establishment and institutionalisation of a catastrophic bond market for agricultural risks in Southern Africa

The institutionalisation of a CAT bond market for agricultural risks in Southern Africa can be a positive step towards hedging climate change risks. Such a market would be very helpful in covering climate change-induced risks, as it mobilises additional private capital: it will bring together sponsors (issuers) and investors (buyers) who transact in an agricultural CAT bond underwritten for a specific climate change risk. In order to regularise these operations, the institutionalisation of the CAT bond market gives provisions on setting up the market as well as how to structure the bond transaction against specific climate change risks.

#### The idea of setting up a catastrophic bond market for agricultural risks in Southern Africa

The stakeholders for the CAT bond market are sponsors, speculative investors and regulating authorities, for example, the Financial Sector Conduct Authority (FSCA) and Johannesburg Stock Exchange (JSE) Debt Market in South Africa, the Namibia Financial Institutions Supervisory Authority (NAMFISA) in Namibia, the Securities Exchange Commission (SEC) and the Insurance and Pensions Commission (IPEC) in Zimbabwe, etc. In principle, an agricultural CAT bond can be structured in the same way as a reinsurance contract. An agricultural CAT bond contract is needed; it will also insulate the bond from coupon and currency (rand) fluctuations. Cumulative principal amounts invested into the agricultural CAT bond and premiums from the sponsor(s) are kept in a collateral account by the SPV, which may invest a part of the funds on the money market to generate short-term returns. Effectively, the SPV becomes a fully collateralised reinsurer with the sponsor(s) as its only client(s), and it is financed by proceeds from the agricultural CAT bond subscribers (investors) (White & Gambrill 2020). Already, this similarity may give an indication that existing agricultural normal loss insurers and reinsurers have a huge role to play in the setting up of the agricultural CAT bond market:

Obviously, the reinsuring SPV will not be bankrupted by any other CAT event than the contractually specified agricultural climate change triggered risks. This gives existing insurers and reinsurers the ability to enhance their underwriting capacity by issuing the CAT bonds – hereby creating an agricultural CAT bond market. Alternatively, insurers and reinsurers may issue CAT bonds on behalf of government or local municipalities to cover national or provincial climate change risks for their agricultural sector. This phase will be the piloting stage.

After the assessment of the CAT bond’s success, the governments, through the respective agricultural ministry and agents, can design an appropriate statutory instrument to launch a Catastrophe Risk Exchange (CARTEX) for climate change risks. This will allow the trading of agricultural CAT bonds not only by the use of SPVs but also on an active secondary market. The CAT bonds may be offered publicly, and speculative investors can bid for them, thereby raising capital to finance climate change risks in the agricultural sector. The concept of such a CARTEX may follow the one used to establish a ‘normal’ stock exchange such as the JSE; private players may be incorporated by means of a public private partnership (PPP) (Steve 2019).

The advantage of using the CARTEX include, inter alia, the ease of generating several intermediaries support who will bring buyers and sellers together while concurrently availing indicative bid and offer spreads on all traded CAT bonds (Risk Management Solutions 2012). Instead of struggling to seek reinsurance, which – in the wake of climate change triggered agricultural risks – may need extra protection from retrocessionaires, a proper institutionalised market for CAT bonds provides an easier and safer option. In addition, the development of a climate linked index (CIX), which may be used to structure agricultural CAT bonds, would provide a further mechanism to secure capital market financing at the CARTEX (Miranda & Farrin 2014; Weber et al. 2015).

Once such a market is set up, the agricultural CAT bonds can be extended to the Southern African region at various levels. In the end, the concept of a CARTEX market would allow for the exchange of climate change risks through subscriptions to a specific CAT bond with other countries, corporates or individuals within or outside Southern Africa.

The adoption of CAT bonds in Southern Africa’s agricultural sector can be done using various alternatives, possibly starting in small jurisdictions to observe their feasibility ahead of a regional roll-out.

#### Alternative ways for setting up a catastrophic bond market for agricultural risks in Southern Africa

Catastrophe bonds have never been used in Southern Africa, but the concept has been employed in Europe and America (Steve 2019; Van Wyk 2021). Transferring it to Southern Africa, in principle, the hedging of climate change risks using CAT bonds can be done at provincial, country/national and regional level with an option of introducing hybrid (all-risks) trigger bids in future.

**Provincial level agricultural CAT bonds:** CAT bonds can be designed to cover provincial-level climate change-related agricultural risks. In this case, the sponsoring insurers can work with municipalities to ensure that climate risks threatening their jurisdiction are hedged against. Parametric CAT bonds are more suitable for this category. For example, the Limpopo province in South Africa or the Matabeleland South province in Zimbabwe can design its own parametric bond against event parameters such as floods coming through the overflow of the Limpopo River. Another example can be of Mozambique’s Cabo Delgrado province, designing a cyclone parametric to cover farming activities in the province as the area has experienced consecutive climate change risks in the past 5 years, such as Cyclone Idai 2019, Tropical Storm Chalane 2020–2021 and Tropical Storm Ana 2022 (Government of Mozambique [GoM] 2022).

**Country-level agricultural CAT bonds:** As most of Southern African countries rely on agriculture for food and nutrition security (FAO 2019), country-specific agricultural CAT bonds may provide sufficient protection to guarantee highest agricultural production. In this case, industry loss agricultural CAT bonds may work best as they guarantee increased cover because the entire insurance industry is aligned to the sponsoring company’s portfolio (RMS 2012). In some cases, indemnity CAT bonds can also work. A country can establish a secondary market where indicative bid and offer spreads on all traded CAT bonds can be provided while deals can be on a matched trade basis.

**Regional-level agricultural CAT bonds:** Agricultural risks can also be regional in scope, as, for example, several countries in the Southern Africa Development Community (SADC) may be affected by a climate change CAT risk/event at the same time. Hence, the effects of climate change may affect more than one country’s agricultural output. This, therefore, affects trade significantly among the countries and also stifles bilateral relations. The adoption of CAT bonds as a new concept leverages the stability and growth of the agricultural sector regionally: if the agricultural risk CAT bond receives sufficient subscribing investors, all the effects of climate change can be hedged. Indemnity CAT bonds and industry loss CAT bonds are much more suitable for hedging regional climate change risk as parametric CAT bonds may be affected by geographical location and territorial trigger limits (Polacek 2018).

**All risks agricultural CAT bonds:** Another option would be to issue an all-risks or hybrid agricultural CAT bond. This will allow all climatic and non-climatic agricultural risks to be covered if they are triggered. This type of CAT bond, however, has disadvantages in attracting speculative investors because, increasing with the number of triggers, the risk of losing a part or even all of the investment principal is very high (Steve 2019). However, it still depends on the CAT bond’s indenture: if professional underwriting skills are used in designing the terms and conditions, even an all-risks CAT bond may work in achieving sustainability in financing agricultural in Southern Africa.

Finally, as a variant, CAT bonds may also be modified in a way that the SPV is given the opportunity not only to invest into more or less risk-free investments but also into the stock market. This would bring in the advantages of diversification as stock market returns can be generated to consolidate the value of the funds held by the SPV in the collateral account. Van Wyk (2021) notes that, by doing structuring modification, CAT bonds can offer higher coverage and higher coupons to investors. To protect not only against the climate change risks but also against the additional risks coming from such investments in non-risk-free assets (but also against movements in the interest rates in case of the short-term financial investments), one may further think about the combination with financial derivates.

### Example: Structuring an agricultural catastrophic bond transaction to hedge against a tropical cyclone and flooding risk

Having discussed the different types of (structuring) CAT bonds and how they might be used in Southern Africa, the following shall exemplify the usage of a catastrophe bond in hedging against a tropical cyclone and flooding risk at South Africa’s Indian Ocean coast:

The South African Civil Protection Department may need to hedge against the flood risk in KwaZulu Natal. The Coast Municipality, as the issuer, may issue a flood CAT bond to the tune of R200m, with a coupon rate of 14% for the 3 years from 01 December 2024 to 30 November 2027. This coupon rate may constitute the money market interest rate (x%), insurance premium (y%) and (small) risk adjustment (z%). The risk adjustment will be small because the insurance premium (y%) is already factored in separately, which may make the risk adjustment small or even nil. The whole coupon rate may be found as follows:

*Coupon rate* = *Money market rate* + *Insurance premium* + *small risk adjustment*

*Suppose, money market rate =* 9*%; insurance premium* = 3%; *small risk adj* = 2%

*Then, Coupon rate*_14%_ = x% + y% + z% = 9% + 3% + 2% = 14%

Investors or sponsors will then subscribe to the bond by paying funds to the Coast Municipality before the commencement of the CAT bond duration on 01 December 2024. These funds will be held in escrow in a secure collateral account either until the risk is triggered or until the maturity of the CAT bond on 30 November 2027.

The structuring of the CAT bond may specify that:

**The investors buy the bond for R200m on 01 December 2024. For reason of simplicity, it shall be assumed that:**

- the index *I* = *f* (*w*, *p*) be expressed just in the monetary damage in a specific currency (say, Rand in this case) and
- the predefined trigger level is set at *I*_Thresh_ = R10m.

**In case a flood occurs, the damage according to the index value *I* will be compared to the predefined trigger level *I*_Thresh_:**

- If damage *I ≤ I*_Thresh_ = R10m, the coast municipality will pay for the damage alone, and the investors will not lose anything.
- If damage *I*_Exh_ = R210m > *I > I*_Thresh_ = R10m, the CAT bond is triggered, and the investors will lose from their invested principal of R200m the portion that is needed to finance the R10m exceeding part for the coast municipality.
  If the invested principal of R200m is not completely exhausted, the CAT bond can be triggered more than once, but the maximum total loss to the investors is capped by their invested principal of R200m:
- If damage *I* ≥ *I*_Exh_ = R210m, CAT bond is exhausted, and the investors will lose their invested principal of R200m completely. The coast municipality will receive these R200m quasi as an insured amount.

**In the case that flood risk is not triggered, the coast municipality will pay the investors R28m on 30 November of each year until 30 November 2027:**

- The R28m will comprise of money market return
  *9% = R18m, insurance premium 3% = R6m and small risk adjustment 2% = R4m*.
- At maturity, that is on 30 November 2027, the investors will also receive their invested principal of R200m back.

**In the case that the CAT bond is triggered, but not exhausted:**

- The investors still receive the interest and the payback, similar as if the flood risk were not triggered, but relative to the remaining capital after paying the coast municipality.

Hence, as long as it is not triggered, the CAT bond is very similar to any other bond as the investors render credit to the coast municipality (in this case: R200m) and receive interest and paybacks at pre-defined dates. However, once it is triggered, the CAT bond serves as an insurance for the municipality, that allows it to cover the R10m exceeding amount of up to R210m. With this money, it can finance the exceeding damages caused by the flood risk such as evacuation costs, temporary shelter provision, food, renovation or reconstruction/replacement costs, among other costs, with any remaining funds being returned to the investor(s) at maturity.

## Managerial implications and research recommendations

The following are the managerial implications and recommendations of this research:

### Managerial implications

Adopting CAT bonds has several managerial implications for all the possible players for such a contract:

- Insurers may increase their underwriting capacities.
- (Speculative) investors may receive higher returns than from other bonds.
- Farmers and agricultural managers can plan proactively for climate change induced risks. Management policies can be informed by this concept and DRM frameworks stand to be guided by this policy, especially in areas farmers struggle to get protection because of insurers’ incapacitation. Therefore, the concept can be useful in achieving sustainable agricultural production despite the effects of climate change.
- The gained safety for production contributes to the increased contribution of the agricultural sector to the gross domestic production (GDP), food and nutrition security in Southern African countries.
- The government may profit from easier coordinating disaster preparedness and risk reduction programmes.
- CAT bonds indirectly enhance underwriting capacity for (re)insurers: as (some, often even severe) risks are transferred from the (re)insurers to private investors, they provide capacity for (re)insurers to accept new risks. Hence, CAT bonds can be used as an alternative risk transfer (ART) instrument that can be combined with traditional reinsurance for climate change risks faced by the agricultural sector.

### Research recommendations

Catastrophic bonds can be structured for specific climate change risks affecting the agricultural sector. This makes it easier for managers to identify triggers and event parameters as well as setting trigger levels that enable effective hedging against climate change risks in agriculture. Thus, CAT bonds can be designed, pretested and adjusted to suit specific climate change risks in specific physical farming areas and agriculture branches such as crop husbandry, animal husbandry, horticulture, aquaculture among related other activities. It is recommended that reinsurers combine CAT bonds with traditional reinsurance to provide larger underwriting capacity for climate change induced risks affecting the agricultural sector. Future research on building climate change resilience using CAT bonds, ART as well as quantitative testing of this CAT bond usage in hedging climate change risks in the agricultural sector should be considered.

## Conclusion

Given that agricultural production in Southern Africa has experienced significant shocks from climate change risks, the adoption of catastrophe bonds (CAT bonds) provides a guarantee that climate risks beyond insurers’ and/or reinsurers’ underwriting capacity will be financed as the capital market returns consolidate insurers’ premiums to fund the agricultural CAT risks. Thus, the underwriting capacities of insurers will be threatened less by the magnitude and severity of climate risks as CAT bonds transfer risks to investors. The capital raised by issuing agricultural CAT bonds lowers insurers’, governments’, municipalities’ or other issuers’ out-of-pocket costs for natural disaster coverage. This means that CAT bonds make it easier to raise capital for climate risks. They may even attract speculative investors as they provide higher coupon rates than other fixed-income securities depending on how they have been structured.

Climate change DRM in the agricultural sector can be significantly enhanced by the use of such bonds as they enhance preparedness for a possible disaster and allow for both proactive and reactive risk reduction as well as spearheading sustainable socio-economic development. In doing so, farmers’ climate change risk management initiatives may be funded to help boost productivity and ensure a sustainable contribution to the gross domestic product in Southern African countries. Therefore, adopting CAT bonds in Southern Africa’s agricultural sectors may assist in addressing capacity and policy gaps around coordinating disaster responses, civil and social protection, humanitarian assistance, agriculture development, management and sustainability, as well as land policies.

The setting up of a CAT bond market for agricultural risks in Southern Africa can bring together all the stakeholders such as farmers, sponsors, speculative investors and regulating authorities together for the smooth flow of CAT bond finances. Considering the rate of climate change and its shocks witnessed in Southern Africa’s agricultural sectors in the past 20 years, the adoption of CAT bond financing may help ensure productivity, food and nutrition security and employment.

## Footnotes

1 It should be mentioned that the characterisation as ‘hybrid’ is ambiguous as literature also uses this attribute to describe a CAT bond that is combined with a financial derivative to protect the bond against volatility in the collateral. For example, Barrieu and Louberge (2009:547) propose to combine ‘the transfer of cat risk with protection against a stock market crash’, employing simultaneously zero-premium digital calls and puts from the index options markets.
